# A qualitative exploration of Ukrainian refugee women’s experiences of homestay accommodation in England: impact on mental health and wellbeing

**DOI:** 10.1016/j.jmh.2026.100456

**Published:** 2026-10-27

**Authors:** Maureen Seguin, Olha Fokaf, Tina Deinekhovska, Aaron Poppleton, Bayard Roberts, Caroline Sanders, Matt Egan

**Affiliations:** a London School of Hygiene & Tropical Medicine, United Kingdom; b World Bank, Ukraine; c Public Partner, United Kingdom; d Keele University, United Kingdom; e University of Manchester, United Kingdom

**Keywords:** Ukrainian refugees, Housing, England, Mental health

## Abstract

In March 2022, the Government of the United Kingdom (UK) introduced two visa schemes enabling persons forcibly displaced from Ukraine to temporarily reside in the UK: the Ukrainian Family Scheme and the Ukraine Sponsorship Scheme (commonly called ‘Homes for Ukraine’). This paper explores experiences of women forcibly displaced from Ukraine living in England under both schemes, highlighting how they perceive their mental health and well-being to have been shaped by their experiences living with hosts. We conducted 55 interviews, of which 39 were women forcibly displaced from Ukraine, 11 were stakeholders, and five were forcibly displaced stakeholders. Stakeholders were persons working at local authorities or other levels of government, volunteers at migration-focused charities, medical practitioners or academics, or translators working within essential services. Interviewees with Homes for Ukraine visa holders revealed a range in host support, spanning paid-for furnished separate housing to households characterised by exploitation and abuse. Many voiced gratitude for supportive hosts yet felt underlying anxiety due to constant impression management and avoiding being an imposition. Accounts by those with Family Scheme visas indicate pervasive overcrowding and a lack of support to secure follow-on accommodation. This lack of security contributed to high degrees of stress as participants attempted to negotiate access to housing support to which they were legally entitled via their visa status. To best support the mental health of forcibly displaced persons in the UK, future homestay initiatives should ensure parity between schemes, sufficient safeguarding and oversight for guests, and improved resources to support host readiness.

## 1 Introduction

‘Homestay’ initiatives, where local households host forcibly displaced persons, have been increasingly implemented in Europe since 2015 (Bassoli and Luccioni, 2024). This accommodation arrangement has been a prominent response to house persons forcibly displaced from Ukraine by the full-scale Russian invasion. In March 2022, the government of the United Kingdom (UK) introduced two schemes to enable persons in Ukraine to enter the UK: The Ukraine Family Scheme (UFS) and the Ukraine Sponsorship Scheme (‘Homes for Ukraine’ or H4U). As of this writing, 58,800 have arrived under the UFS and 159,800 under H4U (for a total of 218,600) since launch (The UK Government, 2024). These schemes can be conceptualised as homestay initiatives, as both involve the hosting of displaced persons in private residences.

The UFS, now closed to new applications, allowed Ukrainians to join family members already in the UK prior to the full-scale invasion. The H4U scheme enables Ukrainian nationals to match with sponsors responsible for providing accommodation for, as a guideline, at least six months. Both schemes allow visa holders to live and work in the UK for up to three years (initially) and access healthcare, housing support and public funds. Analogous schemes were implemented across the European Union and elsewhere, though specific visa routes differ by specific reception, settlement and integration policies (Al-Hamad et al., 2025; Ammann Dula and Fuchs, 2025; Danielsen and Hernes, 2026; Hernes and Łukasiewicz, 2026).

Though the UK response to forcibly displaced Ukrainians has been called ‘generous’ (British Red Cross, 2023; Burrell, 2024), the Ukrainian visa schemes do not offer a pathway to citizenship unlike successful asylum claims (Machin, 2023). Rather, under the original timeframe, UFS and H4U visas were set to expire from March 2025 onwards (three years after being granted). In February 2024, the government announced that the visas were extendable by 18 months, and a two-year extension (in addition to the 18-month extension) was recently announced (McKinney et al., 2025). Visas will start to expire from approximately September 2028 onward, after which visa-holders will be expected to leave the UK pending further policy developments.

Debates over generosity aside (Lewicki, 2022), there is a need to challenge romanticised discourse describing homestay provided for forcibly displaced Ukrainians to access a more nuanced understanding of the experiences of guests (Luczaj, 2023). The H4U scheme has been called a ‘do it yourself’ resettlement program, criticized for reallocating the humanitarian response to Ukrainian refugees from central government to the public with insufficient oversight, risking the exploitation of vulnerable women (Burrell, 2024).

Notable differences in the administration of the UFS and H4U schemes exist. Those entering under H4U were eligible for an initial £200 payment and integration support from their Local Authority (LA). LAs were tasked with conducting security and criminal checks on prospective hosts prior to Ukrainian guests arriving, and inspecting accommodation offered (though notably, not necessarily before guests arrived). H4U hosts received ‘thank-you’ payments of £350 per month, rising to £500 between December 2022 and April 2025 for those hosting for more than one year, then decreasing to £350. LAs received additional funding per H4U visa holder in their jurisdiction. In contrast, UFS visa holders did not receive the initial payment or integration support, their hosts did not receive payments, and their LAs received no additional funding. Accommodation was not inspected, nor were security and criminal checks completed on hosts.

These differences create different risks for visa holders. The lack of housing inspection and lower levels of support from LAs have led some to argue that UFS visa holders may be more vulnerable to homelessness and overcrowding (Machin, 2023). Others have suggested that the financial reimbursement available for H4U hosts may attract hosts with harmful intentions, placing those visa holders at risk for sexual exploitation, abuse and modern slavery (British Red Cross, 2023; Crossley, 2023; Machin, 2023).

Very little has been published on the homestay experiences of Ukrainians under either scheme in the UK. Initial research has outlined the breadth of accommodation which has been provided by hosts under H4U, ranging from spare rooms to entirely separate housing (Berg and Zschomler, 2025; Zschomler and Berg, 2024). Timeframes have been similarly varied, with some hosts adhering to the six-month guideline and others hosting indefinitely. Though most hosting arrangements have been characterised as ‘positive’ (Kuznetsova et al., 2024; Reference removed for anonymity, 2023; Zschomler and Berg, 2024), Berg and Zschomler (2025) observed that Ukrainians found it tiring to be a perpetual guest rather than having their own home. Guests found it difficult to engage in long-term planning without knowing whether their stay in England would be short- or long-term (Zschomler and Berg, 2024). Forcibly displaced Ukrainian women in Reference removed for anonymity (2023) (arriving under both schemes) referenced a loss of independence and feelings of guilt for depending on hosts for shelter and for disturbing hosts’ routines. In several cases, H4U guests lived with hosts less than the six-month guideline because of tension and communication breakdown due to the language barrier and cultural differences.

These findings resonate within the burgeoning literature on experiences of forcibly displaced Ukrainians in homestay accommodation in other high-income countries. Initial positive assessments of homestays are evident across several qualitative studies conducted in Canada (Al-Hamad et al., 2025), Italy (Arcadu et al., 2024), Switzerland (Ammann Dula and Fuchs, 2025) and Poland (Luczaj, 2023). However, inherent challenges are also reported. Over time, Ammann Dula and Fuchs (2025) found that guests became increasingly worried about being a burden on hosts. Due to this worry, and their desire to retain independence, Ukrainian guests sometimes decline help, inadvertently offending hosts (Ammann Dula and Fuchs, 2025). Luczaj (2023) observed that small disagreements were a source of tension, along with mismatched expectations regarding chores. Additional sources of tension from guests’ perspectives include feeling compelled to answer intrusive personal questions and provide favors to hosts (Al-Hamad et al., 2025), amounting to labour exploitation. Emotional exploitation may also be present, where refugee women are expected to show gratitude or conform to hosts’ expectations when they do not wish to do so (Al-Hamad et al., 2025). Both labour and emotional exploitation undermine agency and perpetuate power imbalances between guests and hosts.

A review on homestay accommodation in Europe on publications to 2020 found a relative lack of attention on the views of guests compared with hosts (Bassoli and Luccioni, 2024). Where guests are of focus, they have tended to be young adult males from sub-Saharan countries or the Middle East (Bassoli and Luccioni, 2024). Research focused on homestay initiatives prior to the Ukrainian visa schemes in the UK is sparse, covering the views of unaccompanied refugee teenagers Sirriyeh (2013) and their hosts (Sidery, 2019), and hosts of adult refugees and asylum seekers (Gunaratnam, 2021; Monforte et al., 2021). Sirriyeh (2013) observed that some teenagers felt like part of the family, some like guests in the family, and others like lodgers in their host’s homes. Similar to Al-Hamad et al. (2025); Monforte et al. (2021) noted the emotional exploitation of guests which can occur when guests are reluctant to reveal personal details or return the affection of hosts. Bassoli and Luccioni (2024) note a relative lack of research focus on the role of gender in shaping power relations between hosts and guests.

In their scoping review on homestay accommodation and well-being, Al-Hamad et al. (2024) observed the importance of guest/host dynamics in shaping mental health. Gender dynamics are one of several factors impacting these dynamics, with divergent expectations about gender roles shaping integration experiences for guests (Al-Hamad et al., 2024). Positive interactions, a welcoming environment and a sense of equality between guests and hosts reduced stress and symptoms of anxiety and depression, whilst excessive dependency and overcrowding exacerbated mental health issues (Al-Hamad et al., 2024). Ukrainian guests in Canadian and British homestays have emphasized the importance of mutual cultural exchange, trustful relationships, open communication, and developing reciprocal bonds in creating a sense of home and belonging (Al-Hamad et al., 2025; Berg and Zschomler, 2025). Guest participants in Reference removed for anonymity (2023) shared that living with near-strangers under H4U, or with family in often crowded circumstances led to tension and a yearning for independence. Some felt trapped in unsuitable housing situations due to barriers entering other sorts of housing, leading to feelings of anxiety, helplessness and despair, as well as uncertainty about the future.

### 1.1 Aims and objectives

The aim of this paper is to explore homestay-related experiences of women forcibly displaced from Ukraine in England, with objectives to focus separately on:

1. Those staying with family under the Ukrainian family scheme and
2. Those staying with non-family under the Homes for Ukraine scheme.

Further, we examine:

2. How participants (under both schemes) perceive their mental health and well-being to have been shaped by their homestay experiences.

By addressing these objectives, we offer insights on how future homestay accommodation can be delivered to best support the mental health of forcibly displaced persons.

## 2 Methodology

This is a qualitative study based on in-depth interviews with stakeholders and forcibly displaced women from Ukraine. Stakeholders represented a wide range of professional roles, including staff at LAs and regional and central government, volunteers at migration-focused charities, medical practitioners, academics, and a translator working within the criminal justice system. We interviewed stakeholders as they have a broader view on homestay arrangements and observed mental health impacts than individual forcibly displaced women. The diversity of included roles provides a wide range of vantage points, allowing us to capture relatively rare and/or unusual accommodation arrangements, dynamics with hosts, and mental health outcomes which may have remained unearthed via individual accounts alone. We chose to purposively recruit a Ukrainian translator working in a legal context, which shed light on instances of crimes committed by hosts against guests.

Regarding forcibly displaced participants, we recruited only persons identifying as female, a decision motivated partly by the demographics of Ukrainian adults who have entered in England (The UK Government, 2025c). The decision also reflects research that women and men experience different challenges related to war and displacement differently (Reference removed for anonymity, 2023). This is evidently true amongst the Ukrainian population, with men between 23 to 60 largely prohibited from leaving Ukraine (though exceptions apply). These restrictions have resulted in many families being separated, with many fathers remaining in Ukraine while mothers and children seek refuge abroad. Our focus on women is also in response to the noted paucity of research on forcibly displaced women in homestay accommodation in the UK.

### 2.1 Participant recruitment

Recruitment initially targeted London, Manchester and Liverpool. Male and female stakeholders were recruited from our professional and personal networks within Ukrainian cultural centres in London, Manchester and Liverpool. The highest proportion of H4U visa holders in England is in London (The UK Government, 2025b), and the research team already possessed contacts with the Ukrainian community there through previous work (Reference removed for anonymity, 2023). Manchester and Liverpool were chosen as they host the highest proportions of H4U visa holders outside of southeast England (The UK Government, 2025b), and both cities feature highly-active Ukrainian organisations which facilitated recruitments efforts.

We recruited forcibly displaced Ukrainian women through several approaches. In London, we distributed study leaflets (in Ukrainian) at advice and support sessions provided by LAs. Electronic versions of the leaflet were shared with community-based organisations focused on forcibly displaced Ukrainians, along with LAs based in or near London, Manchester and Liverpool, who cascaded study information through their social medial channels and email lists. We also utilised our personal contacts within the Ukrainian diaspora community to reach potential interviewees. For both interviewee types, we also used snowball recruitment, which facilitated the sample to include participants based beyond London, Manchester and Liverpool.

We endeavored to recruit a diverse sample by age (aged 18 and older), parity (including those with and without children), area of origin in Ukraine, total times displaced (including those initially displaced by the 2014 invasion and subsequently displaced again in 2022), place of residence (covering both the Southeast and Northwest of England) and rurality (covering urban and rural areas).

### 2.2 Data generation

Interview topic guides for forcibly displaced participants focused on the types of housing accessed since arriving in England and their experiences in homestay arrangements (see Appendix A). We probed about interpersonal dynamics in housing arrangements, challenges and benefits related to their accommodation, and whether and how their perceived mental health and/or wellbeing was shaped by their accommodation.

Topic guides for stakeholders focused on their impressions of dynamics between guests and hosts (under both visa schemes), and the administration of the schemes more generally (see Appendix B). For displaced stakeholders, we followed the topic guide for stakeholders but also asked about their personal experiences in homestay. Both types of interviewees received £40 at the end of each interview in recognition of the time taken for the interview.

Interviews were conducted from April to July 2023, apart from two conducted in July 2024 to explore unanticipated findings related to wider study objectives. The first author led the interviews in English, with just under half of the interviews requiring real-time interpretation by coauthors [initials removed for anonymity] and a third Ukrainian interpreter. All interviews were recorded. English-language interviews were professionally transcribed. Those conducted in Ukrainian, Russian, or a mixture of these and English were translated and transcribed by [initials remove for anonymity] and a second translator. English transcripts were cleaned, anonymised and transferred to Microsoft word.

Ethical approval was granted by the [name of ethics board removed for anonymity]. Displaced participants read a Ukrainian version of the participant information sheet and signed a consent form before each interview. Stakeholders read and signed English versions. Information sheets for both participant types stipulated that they could pause or end the interview at any time without giving a reason. Displaced participants were provided with a list of helpful resources at the conclusion of the interview. In anticipation of possible distress caused by interviews, a protocol outlining a course of action was drafted in advance of any being conducted. Though some became emotional during interviews, no one stopped the interview or withdrew from the study. In contrast, many interviewees stated that participating in the interview had been a positive experience.

### 2.3 Analytic method

Analysis was led by the first author. We followed an open coding inductive approach, assigning blocks of text to an evolving set of nodes saved in a Word document. These nodes were then arranged into the larger themes composing the results subsections.

Our approach is informed by elements of reflexive thematic analysis (Braun and Clarke, 2024) and the Big Q Qualitative Reporting Guidelines (Braun and Clarke, 2025). For instance, our organisation of themes in the results section are based on shared meanings (rather than topic summaries), aligned with the concept of ‘artfully interpretive thematic analysis’ (Finlay, 2021). Rather than reporting frequency counts of experiences in the results section, we utilise semi-quantification (Neale et al., 2014). We do not claim to reach saturation, as this term evokes a positivist framing incongruent with our interpretation (Braun and Clarke, 2021). This analytic approach is compatible with our epistemological position of critical realism, which acknowledges both the external reality affecting the lives of participants (for instance, visa schemes which impact access to support) as well as their own subjective perceptions of this reality (Maxwell, 2012).

## 3 Results

### 3.1 Participant characteristics

Fifty-five interviews were conducted, lasting an average 73 min. Of the 55, 39 were conducted with women forcibly displaced from Ukraine, and an additional five were forcibly displaced and stakeholders. The latter had arrived in England under H4U and subsequently commenced work or volunteering activities in LAs and migration-focused charities respectively. Eleven were non-displaced stakeholders and included medical practitioners as well as staff based in regional or central government.

Displaced participants ranged in age from 18–73, with an average age of 41 years old. Thirty-four were parents, with the majority having either one or two children. Two displaced participants had returned to Ukraine by the time of the interview. The remaining 42 lived in Northwest England (n=28) or Southeast England (n=13), with one living in the West Midlands region. Six were living in rural areas. Thirty-seven had H4U visas and six UFS visas. One had entered on a spousal visa, though she would have been eligible for a H4U visa.

Below, we outline experiences of participants accommodated under H4U and the UFS. Threaded throughout accounts are forcibly displaced and stakeholder perceptions on how accommodation experiences shaped mental health and wellbeing. All names are pseudonyms. Consistent with the larger evidence base on homestay accommodation (Bassoli and Luccioni, 2024), we refer below to forcibly displaced participants as ‘guests’ and persons providing accommodation as ‘hosts’ or ‘sponsors.’

### 3.2 Experiences of the homes for Ukraine scheme

Of the 37 participants with H4U visas, 10 were living with hosts (or in separate housing paid by hosts) at the time of the interview. Though most had extremely positive views of their current/previous hosts, some had negative experiences and instances of violence were reported. Drawing as well from stakeholder observations, this span of experiences is outlined below.

#### 3.2.1 “We live as if we are in God's pocket”

Most interviewees who arrived under the Homes for Ukraine scheme shared a roof with hosts and reported good relationships with them. Olha commented, “My sponsors were really nice people. They loved me so much. It was really nice being with them.” Other participants lived in accommodation paid by hosts but separate from the host’s accommodation. For instance, a church community in a small city in Northwest England hosted over 40 displaced Ukrainians, securing, furnishing and paying rent for several properties for an indefinite period. Participants hosted by this group were effusive about the support received. For instance, Oleksandra commented:

When we came, it was beyond our expectations. They rented houses for us that they paid for, filled with furniture. It felt like we stepped into a home, not just a house. There was not just furniture, but baking trays, ice cream scoops, toys for the children, pyjamas with their favorite characters, snuggly blankets, teething cream for the baby, nappies. Prams, bikes. That's how much they gave us. And then they gave us cars. They said ‘these are your cars. Everything in the house is yours. We didn't lend it to you, it's yours.’

She shared that arriving to such a warm welcome made her feel overwhelmed with emotions: “we couldn't believe that someone who didn't even know us would prepare such a house for us to live in. It was amazing. This church is exceptional. People here are exceptional. We live as if we are in God's pocket.”

Many of those who had left their host’s accommodation spoke about them with affection. Polina, a 22-year-old, explained that her hosts had become like ‘second parents’ to her. Though she had returned to Kyiv at the time of the interview, her hosts kept her bedroom as it was and assured her that she would always be welcome to return.

#### 3.2.2 “I didn’t want to impose”

The respect and affection for hosts described above was often mixed with strong feelings of not wanting to impose, especially amongst participants who lived under the same roof as hosts. Single 27-year-old Svitlana remarked “They are the kindest people I have ever met. I love them very much and that is why I wanted to find my own place to make them feel more comfortable.” Though she was not pressured to leave the host’s home, she moved into rented accommodation after about six months because she “did not want to impose.”

Galyna, aged 30 with preschool-aged son, had a good experience with her hosts (in their 60s with grown children), but felt her presence was an imposition. She commented,

They said we could stay as long as we wanted. But I understand that they have their personal life. They have a big family and lots of friends, and sometimes we felt uncomfortable because they wanted to spend time with their children. And then we are also there. They didn't make us feel this way, but I just thought so.

Galyna looked into rental options in England and could have asked her sponsors for help with furnishings. However, she did not want to ask them for more help, as they had already helped her considerably. She decided instead to return to Ukraine.

#### 3.2.3 “I felt I needed to wear a very nice dress and smile and speak about the weather”

Despite the positive comments made about hosts, participants acknowledged that tensions were inevitable especially when two families lived under the same roof. In some cases, participants attributed the tension to the age gap between themselves and their hosts (who tended to older than guests), and the presence of young active children. Victoria arrived in a group of six consisting of her mother, two primary-school aged children, a friend and her daughter, all accommodated within one sponsor’s home. Though she was grateful toward her sponsors and stated “they helped us so much,” she recognised that the arrangement was far from ideal. She recalled, “We had a separate floor but we had only one kitchen and one garden together. We started to feel slightly awkward.” She commented that the trio of children were “very active” and occasionally did naughty things including drawing on a wall and bothering the host’s cat. She put herself in the position of the hosts:

It is their house, they built it, they refurbished it and then six people come with their children drawing on the walls, hitting the cat and breaking glasses. I do understand those were small things but unpleasant ones. If I let six people from a different country into my house, I would behave differently within a week.

Tetyana, who lived with her hosts for just under one year, provided an account of the minutiae of daily scenarios illustrating the difficulty in truly relaxing in host’s homes. She explained,

Can you imagine coming to somebody's house and being a guest for one year? We shared the kitchen and the living room. I get up at 6:00 am every day, and I go to the kitchen to have a cup of coffee. I know that I probably won't see anybody because it's too early, but I can't risk wearing pajamas. So, I need to change my clothes, brush my hair. Just to go to the kitchen and have a cup of coffee. Also, they are vegetarians. I like meat, but I assumed that for them it would not be nice to smell meat. So, I tried to cook meat only when they were away. We had a big TV in the living room and several sofas. And when they were in the living room every evening, we watched the channels they wanted to watch.

Tetyana also alluded to underlying feelings of not being able to be herself; of always needing to be on her ‘best behaviour:’ “I couldn't be myself. I felt I needed to wear a very nice dress and smile all the time and speak about the weather.”

#### 3.2.4 “Some sponsors are terrible people”

A minority of participants recounted experiences with H4U placements characterised by exploitation, abuse, condescension and/or hatred. Stakeholders reported several instances where displaced Ukrainians felt compelled to leave hosts’ homes due to unreasonable rules. Tetyana was a displaced Ukrainian stakeholder working at a LA. Her role involved supporting other Ukrainians arriving under the H4U scheme, which gave her insight into a large range of guest/host dynamics in her area. She had witnessed several examples of guest and hosts ‘falling out’ due to disagreements over house rules. She commented, “some sponsors are terrible people, we have quite a lot of numbers of breakdowns. Some make impossible rules for guests. For example, shower once every two days, wash your clothes only once every two weeks. Use the kitchen only one hour a day.”

Lydia and her primary-school-aged daughter lived with hosts, a couple in their 60s, for almost a year. Tensions arose over the hosts’ condescending and paternalistic attitudes. The hosts implied that Ukraine was a poor, backward country, introducing Lydia to the microwave and clothes washer was when she first arrived. When she started to explore longer-term housing options, the hosts discouraged her from leaving because they did not want her to use benefits paid for by the ‘British taxpayer’ She recounted: “[the host] said, ‘it's *our* money. It's money for the British people. Because we pay taxes. Why should we pay taxes and then you use our tax money?’” Lydia suspected that the hosts also did not want her to leave because they were fond of her daughter, to a point where Lydia felt sidelined and uncomfortable with their influence. They offered to take custody of her daughter, who they treated as a grandchild, as they had a “nice house and could provide her with a good education,” freeing Lydia to return to Ukraine. Further, the hosts made disparaging comments about her mental health to her daughter. She explained, “They were always saying to my daughter ‘you have crazy mom’ Because when we first came, I cried every day because of my brother.” Lydia’s brother was killed in action in March 2022. She identified his body in a large warehouse full of other casualties. These and other traumatic experiences led Lydia to experience symptoms of anxiety and depression. The comments about her mental health led to an argument with the hosts, followed by an eviction notice. At the time of the interview, Lydia was living in temporary accommodation after repeated failed attempts to secure private rental accommodation. She added, “They hate me. I think they hate me. They always tell me about taxes, about the British who help Ukrainian people.”

There was one case of physical violence leading to the end of hosting arrangements, placing 18-year-old Zoryana in a very vulnerable position. She initially got on well with her sponsors, who had a daughter about the same age as her. However, the two teenagers had an argument which escalated into a physical altercation. Zoryana recalled, “We started shouting at each other. The sponsor’s daughter shouted, ‘how dare you shout at me. Who are you here?’” The fight caused some damage to a wall, which the daughter blamed on Zoryana. Zoryana first moved into a hotel room paid for by the sponsor, and then independently made her way to Southeast England to live with a male sponsor she met online. At the time of the interview, she was living in emergency accommodation. She was struggling emotionally with the nature of the hasty departure from her host’s home and was anxious over where she would next live.

The risk of sexual exploitation came up in Tamara’s account. She and her son lived with their host for only a few nights after the LA advised her that the host was a paedophile. He had also purported to have a wife and young children, but when Tamara and her son arrived, he was clearly the only person living there. The pair were relocated into temporary accommodation before being matched with a second host. When asked how these events impacted her mental health at that time, Tamara called it a “bad experience” which was compounded by the fact that she had two brothers who had enlisted around the same time. She recalled, “mentally, I didn’t feel OK. When we arrived here and went through all of this, it’s even worse.”

Though no sexual violence was reported directly by displaced participants, Zoya, a stakeholder of Ukrainian descent who provided interpretation services in police cases recounted one instance. She shared,

There was a rape case committed by a sponsor. There was a man and a woman who lived in quite a lovely massive home. And they invited a Ukrainian refugee and sadly, because she couldn't speak a lot of English, she felt like she had to comply with their orders to clean their house for free as well as other properties that they let out. The male sponsor was raping her for months and she felt that she has no choice but to comply with it because she didn't speak the language. She was far away from anywhere. She didn't know where to go or what to do.

The wife of the abusive host discovered the sexual assaults and, interpreting the behaviour as an ‘affair’, evicted the displaced Ukrainian. The Ukrainian woman contacted the police, which led Zoya’s involvement as the interpreter. She recounted the reluctance of the Ukrainian woman to talk about her experiences: “she felt really ashamed and didn’t want anyone to know about it.” Zoya recalled the male host defended his behaviour by claiming “she never said no.” Zoya recounted that the displaced woman was provided with temporary accommodation and then returned to Ukraine. Zoya speculated there may be more displaced Ukrainian women experiencing sexual violence who were unlikely to report it due to shame and stigma.

### 3.3 Experiences of the Ukrainian family scheme

Six displaced interviewees entered England under the Family Scheme, of which only one was still living with family at the time of their interview.

#### 3.3.1 “I didn’t want to impose”

Like accounts of those participants with H4U visas, a sentiment of not wanting to impose was evident amongst those on the UFS. Yuliya arrived in London with her primary-school-aged son and daughter in September 2022, joining a sister who had lived in London for some time. In their home in Kyiv, each child had their own bedroom, but in London, Yuliya and her two children shared one room. She felt pressure to leave to give her children more space. Also, her sister had a boyfriend, and Yuliya did not want to negatively impact their relationship. She explained, “I don't know her plan, but I don't want to stop her making plans for her own life, with my two kids.” Unemployed at the time of the interview, Yuliya predicted she may return to Kyiv by the autumn of 2023 if she did not manage to find work and a place to live. She speculated, “We are thinking about what we'll do if I do not have a salary. Maybe we'll return to Ukraine. In any case till the end of the July [2023], I still have a goal to be here because of school. Then maybe in the summer, God bless us, everything will finish? I don't know.” Yuliya’s account touched on the negative mental health impact of uncertainty over the future; not being able to predict the length of the war and her stay in England made it difficult for her to make firm housing plans beyond staying with her sister.

#### 3.3.2 “If you arrive under the family scheme the government expects your family to take care of you”

Yulia’s account of overcrowding was common in other accounts of participants with UFS visas. Hanna, a forcibly displaced Ukrainian stakeholder, attributed the overcrowding to the lack of requirement for LAs to check accommodation capacity under the UFS. Petro, a Ukrainian expat stakeholder added “there’s a really big difference in help available under the Homes for Ukraine Scheme and Family Scheme. If you arrive under the Family Scheme the Government expects your family to take care of you.”

Hanna had witnessed countless examples of LAs not stepping in to address overcrowding under the UFS. She shared a scenario where a Ukrainian woman sponsored two sisters to join her in London, each with two children. As the host also had a son, three adults and five children were living in the host’s studio flat. Petro shared an example of an elderly woman sleeping on a sofa at her relative’s home for lack of space: “she is living in their lounge. She doesn’t have a private room and it’s hard for her and I think their relationship has deteriorated.” In both cases, Hanna and Petro appealed to LAs to intervene but were denied support. Petro recounted their response: “the council were like ‘well, there is not much really we can do for her.’” Hanna felt that overcrowding led to relationships between guests and hosts breaking down at a greater rate in the UFS than under the H4U scheme. On the (lack of) LA response, she recounted, “there was an attitude of, if a family invites their relatives, they’re gonna be responsible for their relatives.”

An overall lack of LA support to address overcrowding led to some unusual accommodation arrangements for those on UFS visas. Anastasiya had arrived in England with her husband and primary-school-aged daughter in the early days of the UFS. Facing overcrowded conditions at her relative’s home, she and her family relocated, living on the premises of her husband’s employer. There was no kitchen or washing facilities (other than a sink) in the building. Anastasiya wanted better accommodation but was ineligible for support from her LA and faced barriers entering the private rental market. As such, she felt she had no choice but to continue living on the company premises.

#### 3.3.3 “Earth’s ground was flowing away from under my feet”

Overcrowding sometimes led to eviction threats and abuse of UFS visa holders from family members. Solomiya and her two secondary-school-aged children arrived in late April 2022, joining her cousin’s family in their rented flat in London. Upon arrival, Solomiya was immediately pressured by her cousin’s husband to find alternative accommodation. He threatened that their landlord may evict all of them if they did not leave. She recounted, “I was trying my best but my cousin's husband was always saying that I don't do anything to find a place to live.” Dynamics worsened to the point where he threatened Solomiya with immediate homelessness: “One day, he told me if I won't leave, he will put me, and all my things, in the street.” Luckily, Solomiya had made some contacts in London, one of whom linked her with a lawyer who contacted her LA and facilitated her entry into temporary accommodation.

Alina was also threatened by a family member under the UFS. She arrived with her husband and four children aged 12 and younger in April 2022. She initially lived with her husband’s brother and aged parents, meaning there were six adults and four children living in a three-bedroom apartment. Alina clashed with her mother-in-law, escalating to a point where Alina was forcibly detained in a room by her husband and mother-in-law. She recalled, “I couldn't leave the room for seven hours.” A series of hotel stays for Alina and her children followed, paid out-of-pocket. Arguments over where the children should live escalated to a larger altercation between Alina and her brother-in-law and husband in a Manchester playground. The police became involved, resulting in Alina and her children being taken to a local police station for protection. She recalled, “the children were under a huge amount of stress.” At this point, she felt full of dread, as though “Earth’s ground was flowing away from under my feet.” They were provided with emergency accommodation for one night, but the LA did not initially arrange subsequent accommodation. Alina perceived that she was expected to return to her relative’s home. With limited English, Alina managed to convince the LA that her husband and his family presented a risk to herself and her children, after which the LA provided temporary accommodation. At the time of the interview several months after this upheaval, Alina recounted her fear of her husband: “I was really scared at that point to talk to him.”

## 4 Discussion

This paper makes an important contribution to the literature on experiences of forcibly displaced persons in homestay accommodation. The most striking finding is the disparate experiences of displaced Ukrainian women under the H4U scheme. By essentially placing the public in charge of accommodating this vulnerable group, the support provided is based on differing levels of goodwill rather than standard thresholds. Second, our findings highlight the lack of support from LAs in safeguarding especially UFS visa holders, specifically, addressing overcrowding. Third, it was clear that dynamics within homestays had a large impact on perceived mental health status amongst displaced interviewees. Foregrounding guest perspectives on homestay experiences and interpersonal dynamics (rather than host views) represents a novel contribution to this area of research (Bassoli and Luccioni, 2024).

Most participants arriving under the H4U scheme had a favorable impression of their host yet felt a pervasive stress over not wanting to impose. This complex combination of sentiments resonates with relevant studies in the UK (Reference removed for anonymity, 2023; Zschomler and Berg, 2024) and elsewhere (Al-Hamad et al., 2025; Ammann Dula and Fuchs, 2025; Arcadu et al., 2024; Luczaj, 2023). Some of the H4U participants felt like cherished family members to hosts, similar to some participants in Sirriyeh (2013)’s study on unaccompanied young refugees in the UK. Such positive interpersonal dynamics between hosts and guests appeared to support positive mental health and wellbeing, as also observed in the wider literature (Al-Hamad et al., 2025; Al-Hamad et al., 2024; Berg and Zschomler, 2025). Compared to H4U visa holders, those on the UFS did not commonly describe their hosts in such glowing terms. Due to being related to hosts, perhaps those in the Family scheme had higher expectations of relatives to help them, rendering family support less remarkable than that provided by acquaintances or strangers under the H4U scheme. It is also plausible that hosts under H4U possessed, on average, greater socioeconomic capital than their counterpart hosts in the UFS. Burrell (2024) noted that host interviewees in her study on H4U often (though not exclusively) lived in ‘salubrious’ areas in large homes, an environment incongruent with the overcrowded conditions described by the UFS participants in this study.

In their study on H4U visa holders in England, Berg and Zschomler (2025) observed that forcibly displaced Ukrainians felt it tiring to be a perpetual guest. This paper explores this sentiment further. As also observed by Al-Hamad et al. (2025) participants referenced the emotional labour involved in always presenting oneself (and one’s children) in a favorable way; an inability to completely relax in host’s homes. Participants did not necessarily feel comfortable wearing what they wanted, eating what they wished, and parenting as they desired. The near-constant requirement perceived by women on H4U to be an ideal guest (quiet, tidy, calm, well-behaved) was exhausting and deleterious to mental health and reflect the power asymmetry between guests and hosts.

In some cases, emotional exploitation was evident. Lydia’s hosts implied that Ukraine was a backward country, that she had no right to public funds, that they could provide Lydia’s daughter with a better future than she, and that she was ‘crazy’ Such host/guest dynamics reflects negative stereotypes about Ukrainians and a lack of knowledge about Ukraine from hosts (Galpin et al., 2023) and emotional exploitation, where refugee women are expected to show gratitude or conform to hosts’ expectations and rules when they do not wish to do so (Al-Hamad et al., 2025; Monforte et al., 2021). The consequence of resisting emotional exploitation and negative stereotypes for some guests was the end of homestay arrangements and entries into homeless accommodation (Reference removed for anonymity, 2023).

Some arrangements, under both schemes, ended due to physical or sexual violence or the risk of such violence. Though there were early calls that schemes needed to be monitored to address these risks (British Red Cross, 2023; Machin, 2023), actual accounts of the implications of the lack of such monitoring are largely undocumented in existing research on homestay accommodation. For participants Zoryana and Tamara (under H4U), as well as Solomiya and Alina (under the UFS), these experiences were cited as causing and exacerbating symptoms of poor mental health, worsened still by entries into homelessness due to homestay arrangements ending.

### 4.1 Limitations

We recognise that the experiences outlined here represent only a sliver of experiences of displaced Ukrainian women in England. Our recruitment initially focused on London, Manchester and Liverpool, before branching out into smaller cities and rural locations through snowball sampling. Though this approach led to the inclusion of some residents of smaller cities and rural locations, we do not claim that this paper represents the views and experiences of all Ukrainian visa holders in England. Rural and urban homestay residents faced different challenges due to their environments, which are not fully addressed here. It is possible that guests with particularly negative or positive homestay experiences may have been more likely to participate. We recognise the limited transferability of findings to other parts of the UK, including Wales and Scotland. Those nations operated ‘Super sponsor’ schemes, creating a different hosting landscape to that in England (see Scottish Government, 2026; Welsh Government, 2026).

We acknowledge that the differences observed between the UFS and H4U regarding interpersonal dynamics may be attributed to the timing of interviews in relation to participants’ accommodation arrangements. Most UFS visa holders had already left family members’ homes at the time of their interviews, in contrast to H4U visa holders who were largely still living with hosts. Comments made by the latter of having to constantly engage in impression management, and the emotional labour involved in this activity, may have also been experienced by those arriving under the UFS but were not as pressing on the interview date because they had left their hosting family’s home. It is possible that dynamics between UFS visa holders and family hosts were quite aligned with those shared by H4U visa holders outlined in section 3.2.3. In retrospect, greater effort should have been made to recruit women with UFS visas still living with family hosts to better capture their impressions whilst in the homestay arrangement.

The end of data generation was directed by the study timeline; the need to progress to coding, analysis, and written outputs within a given timeframe. Saturation was not reached, and so the themes identified should not be considered exhaustive of Ukrainian visa holders. New insights and experiences were shared even in the final interviews, reflecting the diverse range in experience across displaced Ukrainian women in England. We recognise this paper does not fully capture this diversity. We believe that additional research focus is required to more fully uncover the benefits and drawbacks of homestay accommodation, so that reception countries can better prepare to accommodate arrivals should this strategy be used again.

### 4.2 Implications for practice

Experiences shared by those on the UFS centered largely on the struggle to access help from LAs to address housing-related issues. As argued elsewhere (British Red Cross, 2023), parity between the UFS and the H4U scheme is necessary to ensure those with UFS visas are provided with the same level of support to their compatriots with H4U visas. Inspection of properties and providing financial assistance to UFS hosts would have created accommodation options more suitable for UFS visa holders, addressing overcrowding in particular.

The striking range in host support under the H4U scheme reflects the ‘do-it-yourself’ nature of the scheme, which resulted in some applicants living in safe homes for an unlimited period and some living in homes where they were assaulted. Increased safeguarding is essential to ensure a threshold quality standard for forcibly displaced persons entering homestay arrangements. Safeguarding could be improved by implementing more stringent criteria by which host suitability is judged. Previous criminal convictions of hosts are detected via a Disclosure and Barring Service (DBS) check, of which there are several types. Currently, LAs have discretion to run ‘basic’ or ‘enhanced’ DBS checks on potential H4U hosts (The UK Government, 2026a), though we acknowledge that the UK Government has outlined scenarios which justify an enhanced check (The UK Government, 2025a). Whilst a basic DBS check will uncover convictions, enhanced checks also include relevant non-conviction information supplied by police forces (The UK Government, 2026c). Stipulating that enhanced checks must be completed on all hosts is justified due to the vulnerable position those on H4U visas are in, especially given the power differential between gusts and hosts.

Our data shows that, in some cases, homestay placements are initially safe but transition into unsafe environments over time. Thus, beyond initial host DBS checks, regular and long-term monitoring of guests is necessary to protect their well-being beyond at the time of their arrival. This requires open communication channels with LAs, as well as adequate resources for LAs to provide support to guests when placements become unsafe or end. Access to adequate housing following homestay arrangements is particularly critical.

The imperative to help Ukrainians leave Ukraine as quickly as possible is cited as a justification for not requiring LAs to complete accommodation checks prior to guests arriving in the UK (National Audit Office, 2023). Whilst this allowed placements to be quickly arranged, our data shows that it also resulted in some unsafe placements (see Tamara’s case above). In future homestay initiatives, we advise that accommodation checks take place before guests arrive to ensure the suitability of the housing and reduce the need to rapidly find alternative accommodation where housing fails checks after arrival.

The UK Government has recently introduced new sponsorship-based routes to facilitate refugees to enter the UK, with arrivals expected from autumn 2027 (Gower, 2026). Though these visa schemes are still in development as of this writing, it is anticipated that sponsors (charities, universities and companies) will provide accommodation and integration support, reducing burden on LAs (The UK Government, 2026b). Nonetheless, LAs will require some level of funding to help run these schemes. As we acknowledge elsewhere (Reference removed for anonymity, 2023), we appreciate that the period of austerity has left LAs with decreased funding for housing and other LA services (Bryan, 2024). This has a negative impact not only on Ukrainian visa holders but anyone in the UK requiring statutory services. Clearly, there are funding challenges which need to be addressed to ensure safe accommodation for those moving on from homestay arrangements, whether these are refugees from Ukraine or elsewhere. As arrivals under the latest sponsorship-based routes are anticipated from autumn 2027, there is a window to consider how to integrate the suggestions above to improve safeguarding of future arrivals both in the UK and beyond.

The spectrum of experiences shared by study participants (under both schemes) suggests areas of improvement in implementation regarding host readiness (see also Al-Hamad et al., 2025). Research on H4U hosts’ needs indicate a desire for assistance with administrative tasks related to hosting, structured guidelines on how to support guests through better signposting to local resources, and greater clarity on how to access accommodation following homestay (Tomlinson et al., 2023). In our data, those guests who appeared to feel the most secure in their hosting arrangements were those whose hosts were the most supportive and well-prepared for their arrival. Host/guest tension was less apparent in those cases where hosts had either a large living space with an adequate number of bedrooms or had the means to rent a separate property for guests. Within our sample, those hosted by a church community (rather than by individuals) appeared to have a high level of satisfaction with their arrangement. This is perhaps due to the larger range of resources available when drawing on a large group of persons supportive of the homestay, versus the resources available from an individual host.

## 5 Conclusion

Placing the UK public in charge of accommodating forcibly displaced Ukrainians has resulted in a huge range of support for this vulnerable population. Some were housed in nurturing environments, characterised by incredibly heart-warming welcomes and unlimited timeframes of financial, housing and emotional support. Though all participants struggled (to various degrees) with the trauma of having fled Ukraine and dealing with the on-going impacts of the war, participants in this environment at least did not have to worry about their housing. On the other end of the spectrum, some participants found themselves in extremely precarious and dangerous housing environments upon arrival in England. Amongst these participants, anxiety over housing was pervasive, compounding trauma due to leaving Ukraine and the war more generally.

Should the homestay model be used in future responses to the arrival of forcibly displaced persons in England, we strongly suggest a higher degree of monitoring and safeguarding to be put in place for those arriving to ensure minimum accommodation standards and an acceptable degree of housing stability. It is critical that LAs are adequately resourced to fulfill these commitments.

## Funding sources

This work was funded by a Three NIHR Research Schools Mental Health Programme Fellowship (award number MH056). The views expressed are those of the author(s) and not necessarily those of the NIHR or the Department of Health and Social Care. The funder had no role in the study design, collection, analysis and interpretation of data, writing of the report and decision to submit the article for publication.

## CRediT authorship contribution statement

**Maureen Seguin:** Writing – original draft, Project administration, Methodology, Funding acquisition, Formal analysis, Data curation, Conceptualization. **Olha Fokaf:** Writing – review & editing, Methodology, Investigation. **Tina Deinekhovska:** Writing – review & editing, Methodology, Investigation, Formal analysis, Data curation. **Aaron Poppleton:** Writing – review & editing, Methodology. **Bayard Roberts:** Writing – review & editing, Supervision, Funding acquisition, Conceptualization. **Caroline Sanders:** Writing – review & editing, Supervision, Funding acquisition, Conceptualization. **Matt Egan:** Writing – review & editing, Supervision, Funding acquisition, Conceptualization.

## Declaration of competing interest

The authors declare that they have no known competing financial interests or personal relationships that could have appeared to influence the work reported in this paper.
