# Supplementary material for: A qualitative exploration of Ukrainian refugee women’s experiences of homestay accommodation in England: impact on mental health and wellbeing
Source: J Migr Health. 2026 Oct 27;14:100456. doi: 10.1016/j.jmh.2026.100456 (PMC13755984; doi:10.1016/j.jmh.2026.100456)
Supplement: Supplementary file 1 [file mmc1.docx]

# Topic guide: Ukrainians in England Interviewee #_______

This is a study on Ukrainian’s access to suitable housing in England. You are likely eligible for housing support from the local council, as well as other services. However, I have heard that there are some barriers in obtaining this help. I am eager to get your thoughts on the housing-related challenges you and other Ukrainians have faced since coming here to England. If I ask anything that you do not want to answer, that is fine, just let me know, and we will talk about something else. We can also stop the interview at any time if you need a break, or if you want to stop participating in the study. You will still be reimbursed £40 for your time taken for the interview if we stop the interview.

## 1. Demographic information

If you are comfortable, can you tell me a bit about yourself?

- Age
- Children? Family composition?
- Marital status
- Previous and current employment/unemployment
- Previous housing in Ukraine
- Visa scheme (Family or Homes for Ukraine)
- Date of entry into UK

## 2. Experiences with housing barriers

If you are comfortable, can you tell me a bit about your current housing situation?

- Live with sponsor?
- Live separately from sponsor, with sponsor paying rent/mortgage
- Has this changed since arriving in UK? Ask about notable developments, build housing chronology.
- Will the current arrangement be changing in future?
  - If so, any plans on what to do next?
  - Barriers – perceived, anticipated, experienced already?

## 3. Impact of housing barriers on access to other services and mental health

Have the barriers related to housing cause barriers in other areas?

- Location of housing dictate school location for children? Educational/language/employment options for adults? Access to health system/GP?
- If you are comfortable doing so, can you tell me whether barriers to accessing suitable housing has affected your mental health and well-being? Has it caused stress?

## 4. Coping with barriers

Can I ask how you are dealing with [follow up on barriers related to housing and other resources mentioned by interviewee]? What do you do to cope?

- Problem solving?
- Seeking support? Details – from charities? Council? Family? Hosts? Online platforms?
- Positive cognitive restructuring – attempting to reframe problems in a more positive light?
- Avoidance? Denial of a problem, engaging in drug or alcohol use?
- Distraction? Focusing on pleasurable activities rather than tackle main issues?

## 5. Addressing housing barriers

If you could change one thing to fix the housing situation in England, what would it be?

- Increase availability?
- Enhance quality?
- Better support from council or charities?
- Affordability?
- Probe about mental health impact – would changes they suggest reduce stress/support mental health and coping?

That’s all the questions I had for you. Do you have anything to add?

[*Ask participant if they know of anyone else who may be willing to be interviewed, point out contact information on information sheet if a potential interviewee wanted to get in touch. Thank the participant, distribute the list of resources and reimbursement of £40, and have them sign a receipt]*
