# Supplementary material for: A qualitative exploration of Ukrainian refugee women’s experiences of homestay accommodation in England: impact on mental health and wellbeing
Source: J Migr Health. 2026 Oct 27;14:100456. doi: 10.1016/j.jmh.2026.100456 (PMC13755984; doi:10.1016/j.jmh.2026.100456)
Supplement: Supplementary file 2 [file mmc2.docx]

# Topic guide: Stakeholders Interviewee #_______

This is a study on Ukrainian refugee access to suitable housing in England. As I understand, [insert councils or name of charity] has facilitated access to secure housing. As such, I am keen to get your perspectives on how the [insert council or charity] has evolved to provide this support, any barriers that you see in Ukrainian refugees accessing this support, and also barriers that Councils may be facing in providing support to Ukrainian refugees. I am also interested in how barriers in accessing secure housing may impact other areas of their lives, such as access to education and employment, and the mental health impact of these barriers.

If I ask anything that you do not want to answer, that is fine, just let me know, and we will talk about something else. We can also stop the interview at any time if you need a break, or if you want to stop participating in the study. You will still be reimbursed £40 for your time taken for the interview if we stop the interview.

## 1. Role and responsibilities

Can you tell me a bit about your current role within the [council or charity], and about your involvement in helping Ukrainian refugees access housing?

- Recollection of events in March – schemes introduced.
- Impacts on the rest of the council structure (staff re-allocated, staffing shortages in home inspection etc) or charity structure?
- Balancing Ukrainian refugee support with other roles?

2. Experiences with housing barriers

Can you tell me a bit about the barriers faced by Ukrainian refugees as they access housing?

- Tensions with sponsors/family members
- Short-term tenure
- Barriers in accessing private rental sector
- Barriers in accessing council homes
- Probe about whether housing challenges have evolved for refugees who arrived in March/April/May 2022.
- Perceptions of housing challenges associated with transition away from Homes for Ukraine scheme – where do Ukrainian refugees go next?
- Perception of future challenges (cliff edge in 2025 when visas expire)?

3. Impact of housing barriers on access to other services and mental health

Have you witnessed barriers related to housing leading to barriers in other areas?

- Location of housing dictate school location for children? Educational/language/employment options for adults? Access to health system/GP?
- Probe about mental health implications of housing barriers

4. Coping with barriers

What are your perceptions on how Ukrainian refugees are coping with these barriers related to housing and other matters?

- Problem solving?
- Seeking support? Details – from charities? Council? Family? Hosts? Online platforms?
- Positive cognitive restructuring – attempting to reframe problems in a more positive light?
- Avoidance? Denial of a problem, engaging in drug or alcohol use?
- Distraction? Focusing on pleasurable activities rather than tackle main issues?

5. Addressing housing barriers

If you could change one thing to fix the housing situation in England, what would it be?

- Increase availability?
- Enhance quality?
- Better support from council or charities?
- Affordability?
- Probe about mental health impact – would changes they suggest reduce stress/support mental health and coping?

That’s all the questions I had for you. Do you have anything to add?

[*Ask participant if they know of anyone else who may be willing to be interviewed, point out contact information on information sheet if a potential interviewee wanted to get in touch. Thank the participant, distribute the list of resources and reimbursement of £40, and have them sign a receipt]*
